# Neurology

**Published:** 1905-04-15

**Authors:** 


(*To be continued.*)

### NEUROLOGY.

**Veronal.**—Burnett[1] has given this new hypnotic in a good many cases with excellent results. The dose is from 5 to 15 grains, an average one being 10 grains. The drug should be dissolved in a hot liquid, as this absolutely disguises the taste and also secures its prompt action. The cases treated were such as are met with in ordinary practice, such as pneumonia, alcoholic insomnia, cellulitis, senile pruritus. No bad after effects were observed in any case and only in one (a severe case of neuralgia) did veronal fail to produce the desired result. It has also been used in cases of maniacal excitement with good results. It is certainly deserving of extensive trial.

**Status Epilepticus.**—Clark and Prout[2] have based a paper on 38 cases of status epilepticus observed in the Craig Colony for Epileptics. They define it as the maximum development of epilepsy, in which one paroxysm follows another so closely that the coma and exhaustion are continuous between seizures. It is almost always accompanied by a marked rise of temperature, with increased frequency of the pulse and respiration indicative of the degree of cerebral exhaustion. The status is usually composed of the major epileptic paroxysms, or rarely it may be composed of one prolonged tonic spasm lasting for an hour or more or until death. It may exceptionally be composed of psychic seizures and vertigo with or without fever. All the superficial reflexes are abolished in this state and the respiration is loud and stertorous. If recovery is to occur the coma is replaced by stupor, and this by delirium and semi-exhaustion, with convalescence within a week. The general rule for basing an unfavourable prognosis is a gradual deliberate increase of the cardinal symptoms, with no modification by sedatives. The best remedy found by experience is potassium bromide 25 grains, chloral hydrate 20 grains, tincture of opium 5 minims, and liquor morphinæ sulphatis 1 drachm, of which one dose is given and repeated in two hours if necessary. The microscopic study of the cerebral cortex in fatal cases shows that the nuclei of the cortical nerve cells undergo disintegration, Nissl granules of the cell bodies disappear, and leave behind a dimly outlined cell framework. The cortex becomes invaded by large mononuclear leucocytes which feed on the débris produced, whilst the neuroglia proliferates to take the place of the destroyed nerve cells. The significance of these changes is important—it shows that the epileptic process is a destructive one.

**Traumatic Apoplexy.**—By this name is understood hæmorrhages into the substance of the brain, essentially different from hæmorrhages into the membranes, and from hæmorrhages which are the immediate and direct results of laceration. According to Bailey[3] there are three varieties of this true traumatic apoplexy—occurring simultaneously with the injury, shortly after the injury, and a long time after the injury. In the first variety the patient, immediately after a blow on the head, develops the symptoms of an apoplexy which may be quickly fatal or produce a hemiplegia. Usually the blow is not severe, and external evidences of injury are wanting. In such cases the evidences of irritation and symptoms of concussion are wanting, whilst signs of general vascular degeneration are present. The explanation of these cases is that there is a pre-existing weakness of the walls of the cerebral arteries, whilst the rise of blood pressure producing rupture is due to fright and excitement rather than to the mechanical effect of the blow. In the second variety the patient, whether made unconscious or not by the blow, quickly recovers and returns to work. After a few days or weeks there begin headache, somnolence and coma, with paralysis of the extremities or of the cranial nerves. The fatal hæmorrhage is generally found to have taken place in the neighbourhood of the fourth ventricle and the aqueduct of Sylvius. Possibly this hæmorrhage is due to local softening. The third variety is generally seen in cases of fracture of the skull with extensive laceration of the brain. The increase in symptoms occurs after a lapse of many years, and is due to a slow increase in the area of thrombosis caused by the injury.

[1] Jour. of Nerv. and Ment. Dis., Dec. 1904. [2] Lancet, Oct. 22 1904. [3] Med. Rec., Oct. 1, 1904.

